# 

**Published:** 1878-12-20

**Authors:** 


☞All communications relating to the business of The Record for the years 1877 and 1878, must be addressed DR. R. C. WORD, Managing Editor Southern Medical Record, Atlanta, Ga.

☞Brief and practical communications are solicited on all subjects pertaining to medicine; also reports of cases in practice.

☞Send money by check, postal order or registered letter.

☞Write your name,post-office, county and State.

RECEIPTED '78 and '79.—L. D. Johnson; to Oct. '79, J. A. Davidson and Geo. S. DeWolf; '78, T. L. Darby, J. W. Herring, W. Rogers, A. B. Loving, J. R. Harrison, E. C. Ellis, A. F. Durham, G. W. Tribble, W. G. Campbell, G. A. Thompson, G. W. Earle. To July, '79, G. L. Glazener, W. R. Jones, J. F. Dorroh, R. J. Gilleland; W. J. Gilliland, '79, W. J. Gilbert, W. J. Rogers, '79; W. C. Robinson, '79.

